# D-dimer as a predictor of cardiovascular outcomes in patients with diabetes mellitus

**DOI:** 10.1186/s12872-022-02531-x

**Published:** 2022-03-04

**Authors:** Lan Cheng, Qianyu Fu, Longhua Zhou, Yuqin Fan, Fenfen Liu, Yuanyuan Fan, Xin Zhang, Weiqing Lin, Xiaohe Wu

**Affiliations:** Jiangxi Provincial People’s Hospital, Nanchang, 330006 Jiangxi China

**Keywords:** D-dimer, Cardiovascular diseases event, Diabetes mellitus

## Abstract

**Objective:**

This study aimed to investigate the association between D-dimer and cardiovascular diseases outcomes in patients with type 2 diabetes.

**Methods:**

This is a single-center retrospective cohort study which was performed in a population who had health examinations between 2010 and 2015 in Jiangxi Provincial People's Hospital. All adult patients who were diagnosed with type 2 diabetes were screened. The cardiovascular diseases events were defined as all-cause mortality, new cardiovascular diseases incidence (acute myocardial infarction, unstable angina, stroke), or cardiovascular mortality.

**Results:**

The median age was 59.6 years; 50.1% of participants were women; D-dimer was significantly associated with endpoint events. After multivariable adjustment for form of treatments and traditional risk factors, the odds ratio was 3.62 (95% CI 2.07–6.03) for the highest quartile of D-dimer with the lowest quartile as reference. Meanwhile, higher D-dimer levels were associated with a significant and independent higher risk of cause-specific cardiovascular disease events.

**Conclusion:**

High plasma concentrations of D-dimer were associated with increased risk of cardiovascular diseases events in patients with type 2 diabetes, even after adjusting for cardiovascular risk factors and form of treatments. Measurement of D-dimer may lead to a practical improvement in the current risk stratification criteria for patients with type 2 diabetes.

## Introduction

Diabetes mellitus is an independent risk factor of cardiovascular diseases. Individuals with diabetes have twofold increased risk of developing cardiovascular diseases compared with normoglycemic individuals [1]. Perfect blood-glucose control substantially decreases the risk of microvascular complications, but not macrovascular disease, in patients with type 2 diabetes [1]. Therefore, it is important to find strategies for preventing caridiovascular dieases in patients with diabetes. Identifying high risk patients is an essential first step in prevention programs. One way of predicting the risk of cardiovascular diseases in high risk patients with diabetes is with biomarkers, such as D-Dimer.

D-dimer is a circulating peptide and degradation product of cross-linked fibrin which is generated from thrombus formation. Higher D-dimer levels reflect more systemic fibrin formation and a tendency for increased thrombosis [2]. It has been reported that high D-dimer concentrations are associated with the cardiovascular diseases events and prognosis [3–5]. However, existing studies included participants who were definitely diagnosed as cardiovascular diseases before recruitments and did not access association of baseline D-dimer levels with cardiovascular diseases events in patients with type 2 diabetes. The current study aims to elucidate the relationship between D-dimer levels and cardiovascular diseases events in a population with type 2 diabetes.

## Subjects and methods

This is a single-center retrospective cohort study including adult patients (≥ 18 years old and < 80 years old) who had health examinations in Jiangxi Provincial People's Hospital from January 1, 2010 to December 31, 2015. Diabetes mellitus was defined as self-reported use of antidiabetic drugs, or a fasting plasma glucose ≥ 7.0 mmol/L, or a 2-h oral glucose tolerance test value ≥ 11.1 mmol/L. According to the diagnosis and classification criteria of diabetes mellitus recommended by Alberti et al. [6], all adult patients who were diagnosed with type 2 diabetes were screened. The exclusion criteria were: type 1 diabetes; gestational diabetes; ketonuria more than 3 mmol/L; serum creatinine greater than 175 µmol/L; retinopathy requiring laser treatment; malignant hypertension; uncorrected endocrine disorder; myocardial infarction, stroke, or unstable angina in the previous year; The clinical endpoints were defined as all-cause mortality, new cardiovascular diseases incidence (acute myocardial infarction, unstable angina, stroke), or cardiovascular mortality. Median follow-up for this cohort was 4.12 years (IQR 2.17–6.01). At entry all patients had a full clinical examination and a fasting blood sample was taken for measurement of biochemical tests and D-dimer. The level of D-dimer was detected using rapid ELISA method. The baseline plasma D-dimer levels were grouped by quartile: ≤ 110 ng/mL; 110–170 ng/mL; 170–270 ng/mL; and ≥ 270 ng/mL. The protocol was approved by the ethics committee of Jiangxi Provincial People’s Hospital. The study was conducted in accordance with the principles of the Declaration of Helsinki. All patients provided written informed consent before enrollment into the study.

## Statistical methods

Baseline characteristics are expressed as the number of observations and percentage for categorical variables or the median and interquartile range for continuous variables. Differences between groups were assessed with the Mann–Whitney *U* test for continuous variables and the χ^2^ test for categorical variables. The D-dimer concentration was analyzed as a categorical variable in quartile. Pearson correlation was used to test correlations between D-dimer levels and traditional cardiovascular risk factors or form of treatments. Univariable conditional logistic regression analyses were used to calculate odds ratios (ORs) and 95% CIs to evaluate associations between baseline D-dimer levels and risk of cardiovascular diseases events. Multivariable conditional logistic regression analyses were adjusted for treatments (anti-thrombotic agents, anti-diabetes agents, and use of statin, ARB/ACEI, β-blocker) and traditional risk factors (hypertension, smoking, body mass index, LDL-cholesterol, triglyceride, HbA1C, duration of diabete, age). And D-dimer in quartile associated with risk of cause-specific cardiovascular disease events were also analysed. SPSS19.0 statistical software was used (IBM SPSS software, Armonk, NY). *P* < 0.05 was statistically significant.

## Results

The present cohort consists of 1976 patients. The median age of the 1976 patients was 59.6 years (IQR 49.7–68.2), nearly half of patients was female (50.1%). The comparisons of characteristics and treatments of patients from CVD events group and non-CVD events group are shown in the Table 1.

**Table 1:** Baseline characteristics in patients with CVD events and without CVD events

Characteristic	Total (n = 1976)	CVD events (n = 214)	Non-CVD events (n = 1762)	*P* value
Age (years)	59.6 (49.7–68.2)	67.4 (51.0–72.4)	56.9 (48.9–67.6)	0.009
Female	989 (50.1%)	102 (47.7%)	887 (50.3%)	0.460
BMI (kg/m^2^)	25.0 (22.9–27.4)	26.4 (23.3–28.9)	24.7 (22.4–26.8)	0.001
Daily smoker	501 (25.4%)	84 (39.3%)	417 (23.7%)	< 0.001
Total-cholesterol (mmol/L)	5.3 (4.2–6.3)	5.4 (4.1–6.2)	5.2 (4.3–6.3)	0.262
HDL-cholesterol (mmol/L)	1.04 (0.64–1.29)	1.04 (0.63–1.29)	1.06 (0.64–1.30)	0.371
LDL-cholesterol (mmol/L)	2.93 (1.25–3.96)	3.87 (2.62–5.42)	2.21 (1.07–3.24)	0.001
Triglyceride (mmol/L)	1.80 (0.94–2.46)	2.13 (1.36–3.93)	1.60 (0.77–2.36)	0.001
Hypertension	881 (44.6%)	127 (59.3%)	754 (42.8%)	< 0.001
HbA1C (%)	6.2 (5.6–7.3)	7.6 (6.3–8.8)	6.1 (5.4–7.0)	0.001
duration of diabetes (years)	6.3 (4.2–9.4)	10.2 (5.7–15.1)	4.6 (3.8–7.9)	0.001
*Treatment*				
Only diet therapy	283 (14.3%)	30 (14.0%)	253 (14.4%)	0.893
Metformin	1245 (63.0%)	103 (48.1%)	1142 (64.8%)	< 0.001
sulphonylureas	563 (28.5%)	88 (41.1%)	475 (27.0%)	< 0.001
Insulin	189 (9.6%)	20 (9.3%)	169 (9.8%)	0.908
asipirin	1116 (56.5%)	97 (45.3%)	1019 (57.8%)	< 0.001
clopidogrel	217 (11.0%)	28 (13.1%)	189 (10.7%)	0.298
warfarin	56 (2.8%)	7 (3.3%)	49 (2.8%)	0.683
statin	1460 (73.9%)	128 (59.8%)	1332 (75.6%)	< 0.001
ACEI/ARB	699 (35.4%)	67 (31.3%)	632 (35.9%)	0.188
β-blocker	211 (10.7%)	29 (13.6%)	182 (10.3%)	0.150
D-dimer (ng/mL)	203 (121–362)	287 (140–407)	197 (118–336)	0.001

Correlations between D-dimer levels and age, sex, form of treatments and traditional cardiovascular risk factors are shown in Table 2. In brief, D-dimer concentrations were positively correlated with age, current smoking, hypertension, body mass index, LDL-cholesterol, triglyceride, HbA1C, duration of diabetes, and negatively correlated with metformin, aspirin and statin therapies.

**Table 2:** Pearson correlation coefficients for correlations between D-dimer levels and cardiovascular risk factors as well as treatments in all patients with CVD events and without CVD events

Risk factor	Correlation coefficient	*P* value
Age (years)	0.14	0.031
Female sex	0.09	0.484
BMI (kg/m^2^)	0.22	0.007
Daily smoker	0.29	0.006
Total-cholesterol (mmol/L)	0.08	0.326
HDL-cholesterol (mmol/L)	− 0.07	0.436
LDL-cholesterol (mmol/L)	0.23	0.019
Triglyceride (mmol/L)	0.18	0.006
Hypertension	0.27	0.001
HbA1C (%)	0.29	0.001
duration of diabetes (years)	0.34	0.001
*Treatment*		
Only diet therapy	0.01	0.462
Metformin	− 0.30	0.013
Sulphonylureas	0.06	0.053
Insulin	0.06	0.072
Aspirin	− 0.33	0.001
Clopidogrel	− 0.003	0.276
Warfarin	− 0.37	0.121
Statin	− 0.12	0.009
ACEI/ARB	− 0.04	0.276
β-blocker	− 0.03	0.391

In a univariable conditional logistic regression analysis, for the D-dimer quartiles, we found an association between the highest quartile of D-dimer and cardiovascular diseases events (OR 3.52; 95% CI 2.14–5.96); with the lowest quartile as reference (data shown in Table 3).

**Table 3:** Univariable and multivariable conditional logistic regression analysis of predictors of cardiovascular diseases events in patients with type 2 diabetes

	Univariable model OR (95% CI)	*P* value	Multivariable model OR (95% CI)	*P* value
Age (years)	1.23 (1.06–1.97)	0.001	1.14 (1.03–1.98)	0.001
Female sex (vs male)	0.87 (0.69–1.33)	0.57		
Normotension	1 (ref)		1 (ref)	
Hypertension	3.3 (2.2–7.4)	0.001	3.19 (2.4–6.3)	0.001
BMI per SD of 2.84 kg/m^2^	1.69 (1.24–3.97)	0.011	1.47 (1.09–3.87)	0.009
Nonsmoker	1 (ref)		1 (ref)	
Daily smoker	1.29 (1.08–1.97)	0.024	1.34 (1.03–2.04)	0.007
Total-cholesterol per SD of 0.95 mmol/L	1.29 (0.65–2.39)	0.423		
HDL-cholesterol per SD of 0.26 mmol/L	0.87 (0.72–1.09)	0.195		
LDL-cholesterol per SD of 1.72 mmol/L	1.56 (1.16–2.93)	0.007	1.64 (1.20–3.07)	0.002
Triglyceride per SD of 0.98 mmol/L	1.24 (1.02–2.69)	0.009	1.29 (1.04–2.37)	0.002
HbA1C per SD of 0.94%	1.69 (1.12–2.65)	0.001	1.64 (1.09–2.87)	0.001
Duration of diabetes (< 5 years)	1 (ref)		1 (ref)	
Duration of diabetes (5–10 years)	1.12 (0.91–1.99)	0.125	1.07 (0.94–2.03)	0.194
Duration of diabetes (10–15 years)	1.77 (1.09–2.73)	0.035	1.62 (1.03–2.86)	0.029
Duration of diabetes (15–20 years)	2.47 (1.76–3.45)	0.001	2.37 (1.84–3.69)	0.001
Only diet therapy	1 (ref)		1 (ref)	
metformin	0.72 (0.54–0.86)	0.001	0.67 (0.55–0.84)	0.001
sulphonylureas	1.16 (1.01–2.34)	0.001	1.12 (1.03–2.76)	0.002
Insulin	1.17 (0.86–1.79)	0.642	1.10 (0.84–1.82)	0.401
aspirin	0.68 (0.42–0.83)	0.001	0.73 (0.44–0.89)	0.001
clopidogrel	0.87 (0.69–1.24)	0.146	0.89 (0.62–1.29)	0.174
warfarin	0.67 (0.40–1.09)	0.061	0.69 (0.42–1.13)	0.070
statin	0.67 (0.41–0.96)	0.001	0.52 (0.39–0.900)	0.001
ACEI/ARB	0.89 (0.77–1.36)	0.125	0.87 (0.79–1.26)	0.109
β-blocker	0.94 (0.86–1.73)	0.067	0.92 (0.81–2.02)	0.093
*D-dimer in quartiles*				
Quartile 1: < 110 ng/mL	1 (ref)		1 (ref)	
Quartile 2: 110–170 ng/mL	1.49 (1.06–2.45)	0.001	1.50 (1.04–2.96)	0.001
Quartile 3: 170–270 ng/mL	2.13 (1.64–3.27)	0.001	2.07 (1.55–3.96)	0.001
Quartile 4: > 270 ng/mL	3.52 (2.14–5.96)	0.001	3.62 (2.07–6.03)	0.001

Multivariable model OR (95% CI) and *P* value adjusted for treatment and traditional risk factors that remained significant after univariable logistic regression analysis: age, hypertension, BMI, smoke, LDL-cholesterol, triglyceride, HbA1C, duration of diabetes, use of metformin, sulphonylureas, insulin, aspirin, clopidogrel, warfarin, statin, ACEI/ARB, β-blocker, D-dimer quartiles

In a multivariable model adjusted for age, hypertension, smoking, body mass index, LDL-cholesterol, triglyceride, HbA1C, duration of diabetes, form of treatments, there also was a significant association between the highest quartile of D-dimer and cardiovascular diseases events (OR 3.62; 95% CI 2.07–6.03); with the lowest quartile as reference (data shown in Table 3).

Higher D-dimer levels were associated with a significant and independent higher risk of cause-specific cardiovascular disease events after adjustment for all significant traditional risk factors and form of treatments. Data (Table 4) shows that a higher D-dimer level was associated with a higher risk of cause-specific cardiovascular disease events during 4.12 years of follow-up within each major category: Q4 versus Q1: for all-cause mortality (OR 1.42; 95% CI 1.29–1.84; *P* = 0.001); for acute myocardial infarction (OR 1.98; 95% CI 1.07–2.66; *P* = 0.001); for unstable angina (OR 1.36; 95% CI 1.04–2.18; *P* = 0.001); for stroke (OR 1.78; 95% CI 1.15–2.09; *P* = 0.001); for cardiovascular mortality (OR 1.42; 95% CI 1.10–2.38; *P* = 0.001).

**Table 4:** Associations between elevated D-dimer levels and cause-specific cardiovascular disease events during 4.12 years of follow-up

D-dimer level (ng/mL)	Events/n	OR (95%CI)	*P* value
*All-cause mortality (n = 69)*			
≤ 110	8/513	1	0.001
110–170	13/502	1.36 (1.19–1.67)	
170–270	17/485	1.30 (1.13–1.66)	
≥ 270	31/476	1.42 (1.29–1.84)	
*Acute myocardial infarction (n = 46)*			
≤ 110	9/513	1	0.001
110–170	10/502	1.24 (1.07–2.04)	
170–270	10/485	1.65 (1.27–2.17)	
≥ 270	17/476	1.98 (1.07–2.66)	
*Unstable angina (n = 50)*			
≤ 110	6/513	1	0.001
110–170	12/502	1.12 (1.04–1.98)	
170–270	13/485	1.27 (1.09–2.03)	
≥ 270	19/476	1.36 (1.04–2.18)	
*Stroke (n = 49)*			
≤ 110	7/513	1	0.001
110–170	9/502	1.30 (1.13–1.89)	
170–270	11/485	1.62 (1.04–2.02)	
≥ 270	22/476	1.78 (1.15–2.09)	
*Cardiovascular mortality (n = 47)*			
≤ 110	4/513	1	0.001
110–170	8/502	1.15 (1.02–1.99)	
170–270	12/485	1.37 (1.09–2.17)	
≥ 270	23/476	1.42 (1.10–2.38)	

OR (95% CI) and *P* value adjusted for treatment and traditional risk factors that remained significant after univariable logistic regression analysis: age, hypertension, BMI, smoke, LDL-cholesterol, triglyceride, HbA1C, duration of diabetes, use of metformin, sulphonylureas, insulin, aspirin, clopidogrel, warfarin, statin, ACEI/ARB, β-blocker, D-dimer quartiles

## Discussion

D-dimer is a soluble degradation product of cross-linked fibrin. Elevated D-dimer levels are found in conditions associated with thrombosis [2]. Therefore, it is usually measured for diagnosing and monitoring venous thromboembolism, pulmonary embolism and disseminated intravascular coagulation [7]. Recently, more and more studies have illustrated that baseline D-dimer level was correlated with cardiovascular diseases incidences and poor prognosis in patients with coronary artery disease [3–5]. Meanwhile, Several studies [8–10] addressed that diabetic patients with microvascular complications had higher D-dimer levels than diabetic patients without microvascular complications. Soares et al. [11] observed that diabetic patients with carotid plaque showed elevated D-dimer levels suggesting that the hypercoagulability state may be involved with the progression of both atherosclerosis and microvascular complications in patients with diabetes [12]. However, there are few studies that access the relationship between D-dimer and cardiovascular diseases events in patients with type 2 diabetes.

The current study found a significant association between baseline D-dimer concentration and the risk of cardiovascular diseases events in patients with type 2 diabetes. This association remained significant after adjusting for confounders, such as hypertension, smoking, body mass index, LDL-cholesterol, triglyceride, HbA1C, duration of diabetes, age, anti-thrombotic agents, anti-diabetes agents, and use of statin, ARB/ACEI, β-blocker. The elevated D-dimer concertration was associated with cardiovascular diseases events independently of conventional risk factors and therapies suggesting that D-dimer measurement should be considered as informative as other conventional risk factors, at least in a diabetes population. The link between D-dimer and cardiovascular disease events is not fully understood. It is reasonable to conceive that early elevation of D-dimer may contribute to the development or the severity of cardiovascular diseases. The impact of higher D-dimer level on cardiovascular diseases events opens the possibility that is implied by the so-called “common soil hypothesis”, which recognizes a number of common mechanisms and risk factors for ischemic vascular disorders [13].

Using confocal microscopy techniques, Alzahrani et al. [14] found that HbA1C level had a correlation with clot structure in patients with diabetes which may probably explain our results showing that D-dimer level had a positive association with HbA1C level and duration of diabetes. Our results supported that poor glycaemic control or prolonged diabetic history may increase thrombotic risk in those population. In a study performed by Sobel et al. [15], they addressed that no matter which treatment strategies was taken, changes in D-dimer level were comparable between insulin-providing treatment strategy and insulin-sensitizing treatment strategy. Nevertheless, in our study, we found that baseline D-dimer level had a negative correlation with metformin treatment and a positive correlation trend with sulphonylureas treatment, though there was not statistically significant, which supported the opinion that the use of insulin-sensitizing drugs conferred more benefit than that of insulin-providing drugs with respect to the balance between fibrinolysis and thrombosis [15]. Unexpected, insulin treatment had neither a association with baseline D-dimer levels nor a association with cardiovascular diseases incidences in diabetic patients. The relationship of blood lipid and D-dimer was also investigated in our study. We found that total-cholesterol and HDL-cholesterol were not significantly correlated with D-dimer. On the contrary, LDL-cholesterol showed an independent positive association with D-dimer. Given it is well known that LDL-cholesterol is a strong independent risk factor of cardiovascular diseases prevalence in whole population, the relationship between D-dimer level and LDL-cholesterol level may support that D-dimer was a predictor not only for cardiovascular diseases events but also for the progression of atherosclerosis in patients with type 2 diabetes. The effect of statin therapy on reducing cardiovascular diseases events appears primarily to be related to the reduction in LDL-cholesterol, although other effects, such as reduced inflammation also appears to play a role [3]. It is well known that anti-thrombotic agents, such as aspirin, clopidogrel and warfarin could reduce D-dimer concentration and cardiovascular diseases events. Nevertheless, in our study, we only found aspirin had a protective effect on cardiovascular diseases events, nor did clopidogrel and warfarin. The possible reason may be the population with type 2 diabetes taken by clopidogrel and warfarin in our study was too small to fit statistic power.

The limitation of our study is the patients enrolled in the study excluded patients with diabetic microangiopathy such as retinopathy or nephropathy. Because there is a hypercoagulability state in diabetes, which may contribute to the progression of microvascular complications and atherosclerosis in diatetic patients [12], meanwhile, macrovascular complications are strongly related to microvascular complications in those, excluding patients with diabetic retinopathy or nephropathy will reduce the significance of the study which try to clarify D-dimer as a predictor of cardiovascular events in diabetic patients.

In conclusion, in a population cohort of type 2 diabetes, elevated D-dimer level at baseline was related to subsequent cardiovascular diseases events from any cause, independently of conventional risk factors or form of treatments. Measurement of D-dimer may lead to a practical improvement in the current risk stratification criteria for patients with type 2 diabetes. Further studies are required to test this hypothesis.

## Data Availability

The datasets used and/or analyzed during the current study are available from the corresponding author on reasonable request.

## Abbreviations

ORs: Odds ratios
CI: Confidence interval
ARB: Angiotensin II receptor blocker
ACEI: Angiotensin-converting enzyme inhibitor
LDL-cholesterol: Low-density lipoprotein cholesterol
HbA1C: Hemoglobin A1c
IQR: Interquartile range
CVD: Cardiovascular disease
HDL-cholesterol: High-density lipoprotein cholesterol

## References

[1] UK Prospective Diabetes Study (UKPDS) Group. Intensive blood-glucose control with sulphonylureas or insulin compared with conventional treatment and risk of complications in patients with type 2 diabetes (UKPDS 33). Lancet. 1998;352(9131):837–53. 9742976

[2] Adam SS, Key NS, Greenberg CS (2009). D-dimer antigen: current concepts and future prospects. Blood.

[3] Simes J, Robledo KP, White HD (2018). D-dimer predicts long-term cause-specific mortality, cardiovascular events, and cancer in patients with stable coronary heart disease: LIPID study. Circulation.

[4] Zhao X, Li J, Tang X (2020). D-dimer as a thrombus biomarker for predicting 2-year mortality after percutaneous coronary intervention. Ther Adv Chronic Dis.

[5] Zhou Q, Xue Y, Shen J, Zhou W, Wen Y, Luo S (2020). Predictive values of D-dimer for the long-term prognosis of acute ST-segment elevation infarction: A retrospective study in southwestern China. Medicine (Baltimore).

[6] Alberti KG, Zimmet PZ (1998). Definition, diagnosis and classification of diabetes mellitus and its complications. Part 1: diagnosis and classification of diabetes mellitus provisional report of a WHO consultation. Diabet Med.

[7] Weitz JI, Fredenburgh JC, Eikelboom JW (2017). A test in context: D-dimer. J Am Coll Cardiol.

[8] Long ZF, Qu GY, Xu M (2001). Relationship between the level of plasma D-dimer and diabetic microangiopathy. Hunan Yi Ke Da Xue Xue Bao.

[9] El Asrar MA, Adly AA, El Hadidy ES, Abdelwahab MA (2012). D-dimer levels in type 1 and type 2 diabetic children and adolescents; relation to microvascular complications and dyslipidemia “own data and review”. Pediatr Endocrinol Rev.

[10] Wakabayashi I, Masuda H (2009). Association of D-dimer with microalbuminuria in patients with type 2 diabetes mellitus. J Thromb Thrombolysis.

[11] Soares AL, Rosário PW, Borges MA, Sousa MO, Fernandes AP, Md C (2010). PAI-1 and D-dimer in type 2 diabetic women with asymptomatic macrovascular disease assessed by carotid Doppler. Clin Appl Thromb Hemost.

[12] Domingueti CP, Dusse LM, Md C, de Sousa LP, Gomes KB, Fernandes AP (2016). Diabetes mellitus: the linkage between oxidative stress, inflammation, hypercoagulability and vascular complications. J Diabetes Complicat.

[13] Di Castelnuovo A, de Curtis A, Costanzo S (2013). Association of D-dimer levels with all-cause mortality in a healthy adult population: findings from the MOLI-SANI study. Haematologica.

[14] Alzahrani SH, Ajjan RA (2010). Coagulation and fibrinolysis in diabetes. Diab Vasc Dis Res.

[15] Sobel BE, Hardison RM, Genuth S (2011). Profibrinolytic, antithrombotic, and antiinflammatory effects of an insulin-sensitizing strategy in patients in the Bypass Angioplasty Revascularization Investigation 2 Diabetes (BARI 2D) trial. Circulation.

